# Prognosis of patients with systemic sclerosis-related interstitial lung disease on the lung transplant waiting list: a retrospective study

**DOI:** 10.1038/s41598-023-37141-w

**Published:** 2023-06-22

**Authors:** Yoichi Nakayama, Ran Nakashima, Tomohiro Handa, Akihiro Ohsumi, Yoshito Yamada, Daisuke Nakajima, Yojiro Yutaka, Satona Tanaka, Satoshi Hamada, Kohei Ikezoe, Kiminobu Tanizawa, Mirei Shirakashi, Ryosuke Hiwa, Hideaki Tsuji, Koji Kitagori, Shuji Akizuki, Hajime Yoshifuji, Hiroshi Date, Akio Morinobu

**Affiliations:** 1 Department of Rheumatology and Clinical Immunology, Graduate School of Medicine, Kyoto University, 54 Kawahara-cho, Shogoin, Sakyo-ku, Kyoto, 606-8507 Japan; 2 Department of Advanced Medicine for Respiratory Failure, Graduate School of Medicine, Kyoto University, Kyoto, Japan; 3 Department of Respiratory Medicine, Graduate School of Medicine, Kyoto University, Kyoto, Japan; 4 Department of Thoracic Surgery, Graduate School of Medicine, Kyoto University, Kyoto, Japan

**Keywords:** Connective tissue diseases, Systemic sclerosis

## Abstract

Advanced systemic sclerosis-associated interstitial lung disease (SSc-ILD) can be treated with lung transplantation. There is limited data on lung transplantation outcomes in patients with SSc-ILD, in non-Western populations.We assessed survival data of patients with SSc-ILD, on the lung transplant (LT) waiting list, and evaluated post-transplant outcomes in patients from an Asian LT center. In this single-center retrospective study, 29 patients with SSc-ILD, registered for deceased LT at Kyoto University Hospital, between 2010 and 2022, were identified. We investigated post-transplant outcomes in recipients who underwent LT for SSc-ILD, between February 2002 and April 2022. Ten patients received deceased-donor LT (34%), two received living-donor LT (7%), seven died waiting for LT (24%), and ten survived on the waiting list (34%). Median duration from registration to deceased-donor LT was 28.9 months and that from registration to living-donor LT or death was 6.5 months. Analysis of 15 recipients showed improved forced vital capacity with a median of 55.1% at baseline, 65.8% at 6 months, and 80.3% at 12 months post-transplant. The 5-year survival rate for post-transplant patients with SSc-ILD was 86.2%. The higher post-transplant survival rate at our institute than previously reported suggests that lung transplantation is acceptable in Asian patients with SSc-ILD.

## Introduction

Systemic sclerosis (SSc) is a chronic autoimmune disorder characterized by microangiopathy and fibrosis affecting the skin and visceral organs. Interstitial lung disease (ILD) is a major complication and the leading cause of death in SSc, accounting for 35% of SSc-related deaths^1^. SSc-associated ILD (SSc-ILD) is a mortality risk factor for patients with SSc^2^. Although recent studies have shown that several immunosuppressants, such as cyclophosphamide, mycophenolate mofetil, and nintedanib (an anti-fibrotic agent), can reduce the rate of decline in forced vital capacity (FVC) in progressive SSc-ILD, there is no evidence-based pharmacological therapy that improves survival and reverses extensive lung damage^3–5^.

Lung transplantation is a potentially life-saving intervention for selected patients with severe ILD refractory to medical therapy^6,7^. Idiopathic pulmonary fibrosis (IPF), a progressive type of ILD, is a widely accepted indication for lung transplantation^7^. However, several centers have been reluctant to offer lung transplants to patients with SSc-ILD and other connective tissue disease (CTD)-associated ILD (CTD-ILD). This is due to concern for worse post-transplant outcomes associated with extrapulmonary CTD manifestations^7,8^. Although recent studies have shown similar outcomes for lung transplantation in patients with IPF and SSc-ILD, more research is needed to determine the safety and efficacy of the transplant approach for SSc-ILD^9–12^.

The 2021 International Society for Heart and Lung Transplantation (ISHLT) consensus document proposed that lung transplantation is an acceptable treatment for selected patients with CTD, including advanced SSc-ILD^13^. However, there are no reports of lung transplantation in patients with SSc-ILD from non-Western countries. Therefore, this study aimed to assess the prognosis of patients with SSc-ILD on the lung transplant waiting list and evaluate post-transplant outcomes for patients attending a lung transplant center in Asia.

## Materials & methods

### Study participants

Using a lung transplant candidate database, patients with SSc-ILD who were registered for deceased-donor lung transplantation at the Kyoto University Hospital between April 2010 and April 2022 were identified. The diagnosis of SSc was made by rheumatologists based on the American College of Rheumatology (ACR) criteria of 1980 or the ACR/European League Against Rheumatism criteria of 2013^14,15^. The registration criteria for the nationwide Japan Organ Transplant Network (JOTN) are: (1) fulfilling the international listing criteria for lung transplantation and (2) age < 60 years for a single lung transplant and < 55 years for a bilateral lung transplant at the time of registration^16–18^. The international listing criteria for lung transplants in patients with ILD are: (1) a rapid decline in pulmonary function over six months, (2) hypoxia, (3) decreased walking distance on the 6-min walk test, (4) pulmonary hypertension (PH), and (5) previous hospitalization due to an acute event (e.g. pneumothorax and acute exacerbation)^16,17,19^. Patients who fulfill one or more of these items are registered on JOTN as candidates for a deceased-donor lung transplant. The algorithm for deceased-donor lung allocation is based primarily on the time the patient has been on the waiting list. In Japan, due to the shortage of donors, single lung transplantation is the standard procedure used for deceased-donor lung transplants for ILDs, including SSc-ILD and IPF. Bilateral deceased-donor lung transplants are warranted only in cases of active chronic respiratory infection, severe PH, and refractory bilateral pneumothorax. Living-donor lung transplants are an option for selected patients with two voluntary donors if the patient cannot continue waiting for a deceased-donor lung transplant due to the severity of their condition^20^.

Patients aged < 18 years at the time of registration and those registered for re-transplantation were excluded from the study. In addition, to investigate post-transplant outcomes, recipients who underwent lung transplantation (deceased or living donor) for SSc-ILD or IPF at Kyoto University Hospital between February 2002 and April 2022 were identified from the lung transplant recipient database.

### Clinical variables

Clinical variables included in the analysis were patient demographics, body mass index, smoking history, distance in the six-minute walk test, SSc subtypes, organ involvement other than ILD, autoantibody profile, the extent of CT involvement, pulmonary function test results (PFT), previous treatments, and long-term oxygen therapy usage. These baseline parameters were obtained at the time of evaluation for lung transplantation. About the evaluation of CT involvement, the extensive disease was defined based on the Goh’s criteria^21^. For other organ involvement, PH was diagnosed if (1) estimated pulmonary artery systolic pressure (ePASP) on transthoracic echocardiogram was 40 mmHg or more or (2) mean pulmonary arterial pressure on right heart catheterization was 25 mmHg or more. Gastrointestinal involvement, such as esophageal dilatation, esophageal motility disorder, or gastroesophageal reflux disease, was screened prior to lung transplantation using upper gastrointestinal endoscopy and high-resolution computed tomography (HRCT), if possible.

### Post-transplant evaluation

All lung transplant recipients underwent PFT at 3, 6, and 12 months post-transplant. They also underwent HRCT pre-transplant and post-transplant at either 3 or 6 months and 12 months. After 12 months, the recipients underwent PFT and HRCT every 12 months. The PFT results at the initial evaluation for a lung transplant and those at 6 and 12 months post-transplant were used to evaluate changes in functional impairment.

HRCT images were reviewed under blind conditions by two observers (TH and KT). Pre-and post-transplant HRCT scans were examined for evidence of relapse in the transplant lungs and changes in the native lungs (deterioration, no change, or improvement). In addition, all available post-transplant HRCT images were examined.

### Statistical analyses

The Mann–Whitney *U* and the Fisher’s exact tests were used for group comparisons. A paired *t* test was used to analyze serial changes in individual patients. Survival time on the waiting list for deceased-donor lung transplants was calculated from the registration date until the patient’s death or living-donor lung transplant. Patients were right-censored at the time of the deceased-donor lung transplant or the last contact. The last observational date was 30 April 2022. A living-donor lung transplant was counted as an event as it represented the emergence of a fatal condition. The cox proportional hazards regression analysis was used to identify the factors predictive of mortality on the waiting list. The post-transplant survival time was calculated from the date of the lung transplant to the patient’s death, with patients right-censored at the date of re-transplantation (including the second transplantation for the residual native lung) or the last contact prior to 30 April 2022. Kaplan–Meier curves and the log-rank test were used to demonstrate and compare the survival time post-transplant between the SSc-ILD and IPF groups. Statistical analyses were performed using EZR (Saitama Medical Center, Jichi Medical University, Saitama, Japan), a graphical user interface for R (The R Foundation for Statistical Computing, Vienna, Austria)^22^. All analyses were two-tailed, and a *P*-value of < 0.05 was considered statistically significant.

### Ethics

All study participants provided informed consent, and the study design was approved by the appropriate ethics review board. Therefore, this observational study (not being a clinical trial) was conducted in accordance with the Declaration of Helsinki and approved by the institutional review board (Kyoto University approval number R1355, R1353, R2401).

## Results

### Characteristics and prognosis of patients with SSc-ILD registered for deceased-donor lung transplantation

Twenty-nine patients with SSc-ILD registered for deceased-donor lung transplants between April 2010 and April 2022 (Table 1). Of these, ten patients received deceased-donor lung transplants (34%), two received living-donor lung transplants (7%), seven died while waiting for a lung transplant (24%), and the remaining ten survived on the list (34%) (Supplementary Fig. S1). The median waiting time from registration to deceased-donor lung transplant was 28.9 months (range 22.3–30.3). The median duration from registration to living-donor lung transplant or death (events) was 6.5 months (range 4.1–14.7).

**Table 1:** Demographic characteristics of systemic sclerosis interstitial lung disease patients registered on lung transplantation waiting list.

	Patients (N = 29)
Age, mean (S.D.)	47.2 (9.5)
Female, n (%)	18 (62)
Disease duration (Y), median (IQR)	7.0 [5.1, 12.3]
BMI (kg/m^2^), median (IQR)	21.9 [19.4, 24.8]
(Ex-)smokers, n (%)	16 (55)
6MWD (m), median (IQR)	355 [251, 485]
SSc subtypes	
lcSSc, n (%)	14 (48)
dcSSc, n (%)	15 (52)
Symptoms/complications of SSc	
Raynaud phenomenon, n (%)	27 (93)
Digital ulcer, n (%)	2 (7)
PH, n (%)	14 (48)
GI involvement^a^, n (%)	20 (69)
SRC history, n (%)	1 (3)
Treatment	
PSL, n (%)	25 (86)
PSL dose (mg), median (IQR)	10 [5, 15]
Calcineurin inhibitors, n (%)	10 (34)
IVCY (previous use), n (%)	11 (38)
Nintedanib, n (%)	6 (21)
Autoantibodies	
ACA, n (%)	3 (12)
ATA, n (%)	15 (56)
RNA polymerase-III, n (%)	0 (0)
U1-RNP, n (%)	4 (15)
Extensive CT involvement^b^, n (%)	22 (76)
Spirogram	
% FVC, median (IQR)	51.1 [44.7, 57.4]
% DLCO, median (IQR)	28.6 [21.8, 35.1]
LTOT, n (%)	23 (79)
Outcomes	
Deceased-donor transplant	10 (34)
Living-donor transplant	2 (7)
Died awaiting transplant	7 (24)
Alive on the waiting list	10 (34)

*6MWD* Six-minute walk distance, *ACA* anti-centromere antibody, *ATA* anti-topoisomerase-1 antibody, *dcSSc* diffuse cutaneous systemic sclerosis, *DLCO* diffusing capacity of the lungs for carbon monoxide, *FVC* forced vital capacity, *GI* gastrointestinal, *ILD* interstitial lung disease, *IQR* interquartile range, *IVCY* intravenous cyclophosphamide, *lcSSc* limited cutaneous systemic sclerosis, *LTOT* long-term oxygen therapy, *PH* pulmonary hypertension, *PSL* prednisolone, *RNA* ribonucleic acid, *RNP* ribonucleoprotein, *S.D*. standard deviation, *SRC* scleroderma renal crisis, *SSc* systemic sclerosis, *Y* year/s.

^a^Gastrointestinal involvement included esophageal dilatation, esophageal motility disorder, and gastroesophageal reflux disease detected using gastrointestinal endoscopy or computed tomography.

^b^Extensive CT involvement was defined based on the Goh’s criteria (the total disease extent in high-resolution CT > 20% was determined as ‘extensive’).

Of the patients with SSc-ILD registered for a deceased-donor lung transplant, characteristics of the patients who received a deceased-donor lung (n = 10), a living-donor lung, or those who died on the waiting list (n = 9) are compared in Table 2 to identify associated factors of a worse outcome. PH was more common in patients receiving living-donor lungs and those who died on the waiting list. It was also associated with mortality and switching to living-donor lung transplantation while waiting for a deceased-donor lung (hazard ratio: 10.1; 95% confidence interval: [1.26–81.1], *P* < 0.01) (Table 3).

**Table 2:** Characteristics of systemic sclerosis-related interstitial lung disease patients with deceased/living-donor or who died awaiting transplant.

	Deceased-donor lung transplant (N = 10)	Living-donor lung transplant or died awaiting transplant (N = 9)	*P* value
Age, mean (S.D.)	45.8 (3.1)	47.8 (3.3)	0.67
Female, n (%)	6 (60)	6 (67)	0.76
Disease duration (Y), median (IQR)	6.4 [1.2, 13.1]	8.0 [4.0, 9.7]	0.46
BMI (kg/m^2^), median (IQR)	21.5 [20.5, 24.0]	21.2 [15.3, 22.8]	0.41
(Ex-)smokers, n (%)	7 (70)	5 (56)	0.51
6MWD (m), median (IQR)	342 [251, 459]	263 [151, 305]	0.16
SSc subtypes, lcSSc(n)/dcSSc(n)	6/4	5/4	0.84
Symptoms/complications of SSc			
Raynaud phenomenon, n (%)	10 (100)	9 (100)	NA
Digital ulcer, n (%)	1 (10)	1 (11)	0.93
PH, n (%)	3 (30)	8 (89)	0.009
GI involvement^a^, n (%)	7 (70)	6 (67)	0.88
SRC history, n (%)	1 (10)	0 (0)	0.33
Treatment			
PSL, n (%)	8 (80)	9 (100)	0.16
PSL dose (mg), median (IQR)	10 [3, 15]	10 [6, 15]	0.66
Calcineurin inhibitors, n (%)	5 (50)	2 (22)	0.21
IVCY (previous use), n (%)	3 (30)	4 (44)	0.51
Nintedanib, n (%)	0 (0)	1 (11)	0.28
Autoantibodies			
ACA, n (%)	0 (0	1 (13)	0.33
ATA, n (%)	5 (50)	2 (25)	0.20
RNA polymerase-III, n (%)	0 (0)	0 (0)	NA
U1-RNP, n (%)	2 (25)	2 (22)	0.89
Extensive CT involvement^b^, n (%)	9 (90)	7 (78)	0.47
Spirogram			
%FVC, median (IQR)	56.6 [52.9, 65.7]	47.8 [34.9, 55.6]	0.10
%DLCO, median (IQR)^c^	23.7 [21.3, 25.0]	33.9 [18.4, 36.1]	0.46
LTOT, n (%)	9 (90)	9 (100)	0.33
Duration from registration date to outcome date (M), median (IQR)	28.9 [22.3, 30.3]	6.5 [4.1, 14.7]	< 0.001

*6MWD* Six-minute walk distance, *ACA* anti-centromere antibody, *ATA* anti-topoisomerase-1 antibody, *dcSSc* diffuse cutaneous systemic sclerosis, *DLCO* diffusing capacity of the lungs for carbon monoxide, *FVC* forced vital capacity, *GI* gastrointestinal, *IQR* interquartile range, *IVCY* intravenous cyclophosphamide, *lcSSc* limited cutaneous systemic sclerosis, *LTOT* long-term oxygen therapy, *M* month/s, *PH* pulmonary hypertension, *PSL* prednisolone, *RNA* ribonucleic acid, *RNP* ribonucleoprotein, *S.D*. standard deviation, *SRC* scleroderma renal crisis, *SSc* systemic sclerosis, *Y* year/s.

^a^Gastrointestinal involvement included esophageal dilatation, esophageal motility disorder, and gastroesophageal reflux disease detected using gastrointestinal endoscopy or computed tomography.

^b^Extensive CT involvement was defined based on Goh’s criteria (the total disease extent in high-resolution CT > 20% was determined as “extensive”).

^c^Because of low vital capacity, there were three missing data points from the deceased-donor lung transplant group and four missing from the living-donor lung transplant or died awaiting transplant group.

**Table 3:** Risk factors for death or living-donor transplantation while awaiting deceased-donor lung transplantation.

	Univariate cox model		
	HR	95% CI	*P* value
Age	1.01	0.95–1.09	0.82
Sex, male	0.65	0.16–2.60	0.53
BMI	0.85	0.71–1.00	0.056
6MWD (m), median (IQR)	0.99	0.98–1.00	0.054
PH	10.1	1.26–81.1	0.0049
% FVC	0.97	0.93–1.03	0.31
% DLCO	1.00	0.89–1.11	0.99

*6MWD* Six-minute walk distance, *BMI* body mass index, *CI* confidence interval, *DLCO* diffusing capacity of the lungs for carbon monoxide, *FVC* forced vital capacity, *HR* hazard ratio, *PH* pulmonary hypertension.

### Outcomes of lung transplantation for patients with SSc-ILD

Fifteen patients received a lung transplant for SSc-ILD between February 2002 and April 2022; three patients underwent a deceased-donor bilateral lung transplant, seven underwent a deceased-donor single lung transplant, four had a living-donor bilateral lung transplant, and one had a living-donor single lung transplant (Supplementary Fig. S1). In addition, three patients received a living-donor lung transplant without registration for a deceased-donor lung transplant. Individual lung transplant cases are summarized in Table 4. All recipients were managed with a combination of corticosteroids (maintenance dose: prednisolone 0.2 mg/kg every two days), calcineurin inhibitors (cyclosporine A or tacrolimus), and mycophenolate mofetil as the standard regimen of post-transplant immunosuppression.

**Table 4:** Description of systemic sclerosis-related interstitial lung disease patients who underwent deceased- or living-donor lung transplantation.

No	Age	Sex	Autoantibody	Transplant type	Wait time (mo)	PH	GI involvement^a^	LTOT pre-transplant	Outcomes	Follow-up post-transplant (mo)	LTOT post-transplant	ILD relapse in transplanted lungs	ILD exacerbations in residual lungs
1	42.7	M	ANA, ATA	Deceased (B) (due to PH)	30	+	+	+	Alive	71	–	–	(No residual)
2	25.4	F	ANA	Deceased (B) (due to recurrent bilateral pneumothorax)	29	–	+	+	Alive	58	–	–	(No residual)
3	52.9	F	ANA, ATA, U1-RNP	Deceased (B) (due to chronic lung infection)	30	–	+	+	Dead (sepsis)	62	(Dead)	–	(No residual)
4	61.9	F	ANA	Deceased (S)	28	–	+	–	Alive	26	–	–	–
5	58.7	F	ANA, U1-RNP	Deceased (S)	17	+	–	+	Alive	96	+	–	+
6	31.8	F	ANA, ATA	Deceased (S)	18	–	+	+	Re-transplantation for bilateral lungs (living-donor (B))	47	(Retransplant)	–	–
7	48.1	M	ANA, ATA	Deceased (S)	30	+	+	+	Alive	42	–	–	–
8	54.7	M	ANA	Deceased (S)	25	–	+	+	Dead (CLAD)	25	(Dead)	–	+
9	49.2	F	ANA	Deceased (S)	25	–	+	+	Alive	31	–	–	–
10	59.3	M	ANA, ATA	Deceased (S)	37	+	+	+	Alive	47	–	–	– (improved)
11	59.4	M	ANA	Living donor (B)	–	+	+	+	Alive	86	–	–	–
12	54.8	F	None	Living donor (B)	–	+	–	+	Alive	163	–	–	(No residual)
13	57.8	F	ANA	Living donor (B)	4	–	–	–	Dead (sepsis)	8	(Dead)	–	(No residual)
14	56.0	F	ANA, ACA	Living-donor (B)	11	+	+	+	Alive	8	–	–	(No residual)
15	43.8	F	ANA, ATA	Living donor (S)	–	+	–	+	Re-transplantation for the contralateral native lung (deceased (S))	35	(Retransplant)	–	+

– absent, + present, *ACA* anti-centromere antibody, *ANA* anti-nuclear antibody, *ATA* anti-topoisomerase I antibody, *B* bilateral lung transplantation, *CLAD* chronic lung allograft dysfunction, *F* female, *GI* gastrointestinal, *ILD* interstitial lung disease, *LTOT* long-term oxygen therapy, *M* male, *mo* month/s, *PH* pulmonary hypertension, *RNP* ribonucleoprotein, *S* single lung transplantation.

^a^Gastrointestinal involvement included esophageal dilatation, esophageal motility disorder, and gastroesophageal reflux disease detected using gastrointestinal endoscopy or computed tomography.

During the median post-transplant observation time of 47 months (range 26–71), three of the fifteen lung transplant recipients with SSc-ILD died (20%). One patient died of chronic lung allograft dysfunction (CLAD), and two died of sepsis. Two patients received a second lung transplant after a single lung transplant. One patient (patient No.6) developed CLAD after a deceased-donor lung transplant. They underwent a further living-donor bilateral lung transplant 47 months after their initial transplant. Another patient (patient No.15) developed CLAD in their transplanted lung and had disease progression in their native lung. They underwent a deceased-donor right lung transplant 35 months after their initial living-donor left lung transplant. This patient had undergone their initial living-donor single lung transplant as an emergency measure as a deceased-donor lung was unavailable. Three patients in total were diagnosed with CLAD (20%). The estimated 1-year and 5-year post-transplant survival rates (re-transplantation-censored) from the time of lung transplantation were 93.3% and 86.2%, respectively. Post-transplant survival was similar in the bilateral and single lung transplant groups (log-rank test, *P* = 0.46).

**Figure 1:**
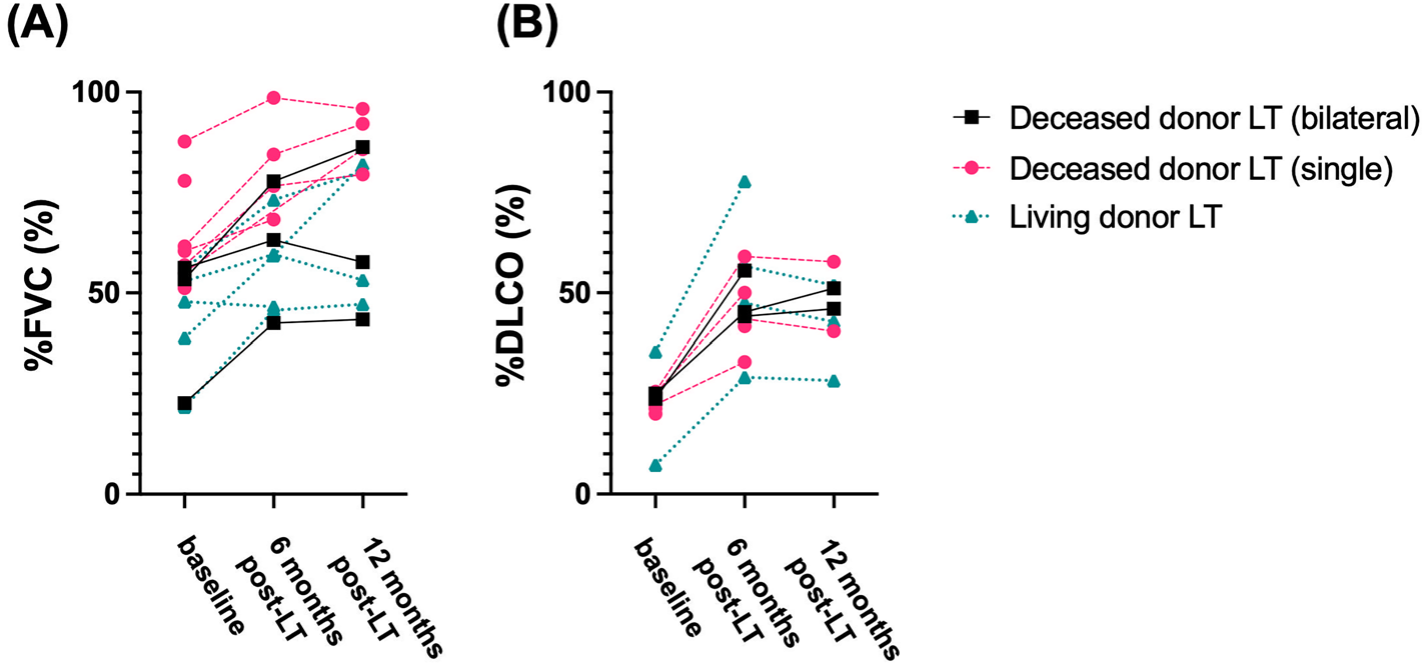
(**A**) Changes in forced vital capacity (%) from the baseline (pre-transplant) to 6 and 12 months post-transplant. *FVC* forced vital capacity, *LT* lung transplantation. (**B**) Changes in diffusing capacity of the lung for carbon monoxide (%) from the baseline (pre-transplant) to 6 and 12 months post-transplant. *DLCO* diffusing capacity of the lung for carbon monoxide.

The PFT results from 6 and 12 months post-transplantation were obtained for 15 patients. Changes in %FVC from pre-transplant to 6 and 12 months post-transplant are shown in Fig. 1A. In all patients, the median %FVC was 55.1% (interquartile range [IQR] 47.8–60.4%) pre-transplantation and improved to 65.8% (IQR 49.8–77.5%) at 6 months and 80.3% (IQR 53.2–86.3%) at 12 months post-transplant. Nine patients (69%) were free from long-term oxygen therapy use 6 months post-transplant. Changes in % diffusing capacity of the lung for carbon monoxide (DLCO) from pre-transplant to 6 and 12 months post-transplant are shown in Fig. 1B. In all patients, the median % DLCO was 23.7% (IQR 20.7–25.3%) pre-transplantation and improved to 46.4% (IQR 42.1–56.4%) at 6 months and 46.0% (IQR 40.5–51.9%) at 12 months post-transplant.

The pre-transplant and post-transplant data of ePASP on transthoracic echocardiogram were obtained for patients who had pulmonary hypertension before LT. The median ePASP was 56 mmHg (IQR 46-77 mmHg) before LT and decreased to 37 mmHg (IQR 24-41 mmHg) at six months post-transplant.

During the post-transplant period (median 47 months, range 26–71), there was no ILD relapse in the transplanted lungs. A renal crisis was not observed for any patient. Disease progression in the native lung on HRCT was observed in three of the eight single lung transplant recipients.

### Comparison of post-transplant outcomes between patients with SSc-ILD and IPF

**Figure 2:**
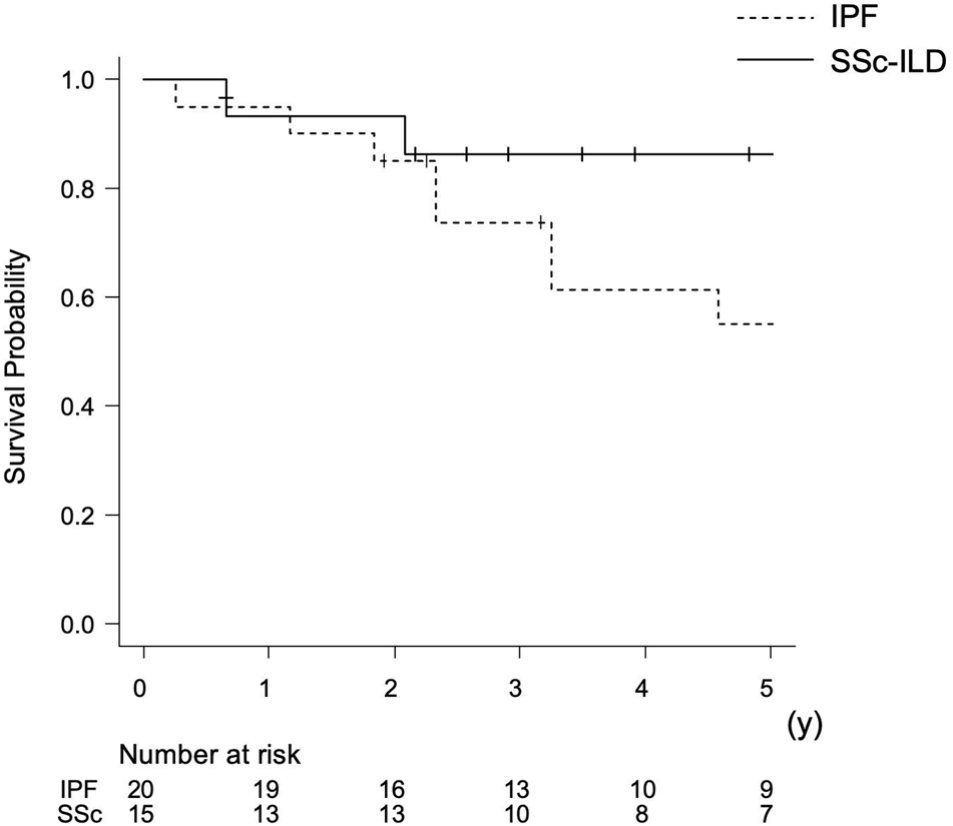
The estimated 5-year post-transplant survival curves (re-transplantation-censored) post-transplant in patients with systemic sclerosis-related interstitial lung disease and idiopathic pulmonary fibrosis. *IPF* idiopathic pulmonary fibrosis, *SSc*-*ILD* systemic sclerosis-related interstitial lung disease.

Fifteen patients with SSc-ILD and twenty with IPF (diagnosed through multidisciplinary discussions) underwent lung transplantation between February 2002 and April 2022. Of the 20 cases of IPF, 11 (55%) were deceased-donor single lung transplants, and nine (45%) were living-donor bilateral lung transplants. The baseline characteristics of recipients with SSc-ILD and IPF are summarized in Supplementary Table S1. Patients in the IPF group were less likely to be female than male, had a lower incidence of PH, and had a lower prednisolone use rate. During the post-transplant period (median 47 months, range 26–71), eight of the twenty recipients with IPF died, and none received re-transplantation. The estimated 5-year post-transplant survival rate (re-transplantation-censored) was 55.3% in the IPF group and 86.2% in the SSc-ILD group (*P* = 0.33) (Fig. 2).

## Discussion

In this study, 34% of patients with SSc-ILD on the waiting list received a deceased-donor lung transplant after a median wait time of 28.9 months. A total of 31% of patients on the wait list died or required a living-donor lung transplant as an emergency treatment. Lung transplantation improved pulmonary function and led to the cessation of long-term oxygen therapy in approximately 70% of patients, with no relapse of SSc-ILD in the transplanted lungs and no serious deterioration of extrapulmonary manifestations post-transplant. Post-transplant survival for patients with SSc-ILD was comparable to those with IPF.

To the best of our knowledge, this study is the first to evaluate the outcomes of patients with SSc-ILD registered for deceased-donor lung transplants. Similar studies of patients with IPF on the waiting list for deceased-donor lung transplants showed that 40–60% of patients died while awaiting transplants^23–27^. We recently reported that in a study of 166 patients on the transplant waiting list (who had ILDs, including SSc-ILD, IPF, and other ILDs), 33% of these patients received a deceased-donor lung transplant^19^. Another study from a single institute in Japan showed lower mortality rates for patients with non-IPF ILD (57.9% of patients with CTD) than those with IPF, 40.4% vs 61.4%, respectively^27^. Our results (deceased-donor lung transplant in one-third of patients and mortality or a living-donor lung transplant in another third) were comparable with the reported outcomes of patients with IPF and other ILDs on the waiting list for a deceased-donor lung transplant in Japan. Thus, registration for deceased-donor lung transplants should be considered for patients with severe SSc-ILD at an appropriate time, as well as for those with IPF and other ILDs.

Approximately one-third of patients with SSc-ILD died or needed living-donor lung transplants as an emergency treatment, if available, during the waiting time for a deceased-donor lung transplant. PH was predictive of these fatal outcomes. The combination of ILD and PH in SSc has been associated with a worse prognosis than ILD or PH alone^28,29^. The 2021 ISHLT consensus document proposed three pulmonary phenotypes of SSc in lung transplant candidates according to the extent of ILD and hemodynamic profiles: predominant ILD, combined ILD-PH, and predominant PH^13^. Earlier waitlisting for a deceased-donor lung transplant may be advisable if a patient with SSc has a combined ILD-PH phenotype.

In 2021, the ISHLT proposed lung transplantation was an acceptable option for selected patients with advanced CTD-ILD, including SSc-ILD, after a long-standing debate lasting decades^13^. Particular concerns for patients with SSc-ILD include an increased risk of CLAD associated with esophageal dysmotility and, consequently, a worse post-transplant survival rate than other ILDs^16,30^. However, high-volume lung transplant centers in both the US and Europe have consistently reported acceptable outcomes of lung transplantation for SSc,the estimated 1-year survival rates post-transplant were 81–100%, and the 5-year survival rates were 61–76%^9–11,31–33^. The risk of CLAD was similar between lung transplants for SSc and those for other ILDs. Esophageal disease in SSc did not affect post-transplant survival compared with lung transplants for recipients with other ILDs^11,31,32^. Our results are consistent with previous reports from the US and European lung transplant centers and support the 2021 ISHLT proposal. This study is the first lung transplantation report in cases of SSc-ILD from a high-volume center in Asia. The estimated 5-year post-transplant survival rate was higher than previous reports from other countries, suggesting that favorable post-transplant outcomes can also be achieved in non-Western high-volume lung transplant centers.

The potential effects of the underlying autoimmune condition on the transplanted tissue have been postulated for SSc and other CTD-ILDs^31,33^. In our study, there was no relapse of ILD in the transplanted lung, although ILD in the native lung was progressive for some patients who received a single lung transplant. No serious extrapulmonary complications associated with SSc, such as renal crisis or bowel pseudo-obstruction, were noted. The 2021 ISHLT consensus document listed cardiac, venous thromboembolism, renal (renal crisis), gastrointestinal, and vascular (Raynaud’s phenomenon) involvement as disease-specific extrapulmonary manifestations that require consideration and evaluation prior to lung transplantation for SSc^13^. Although the previous 2014 ISHLT criteria for lung transplantation did not include disease-specific listing criteria for SSc-ILD^16^, patients with either active or uncontrolled extrapulmonary manifestations, severe swallowing/esophageal dysfunction, and active myocarditis were excluded from the registry for lung transplantation. Thus, if transplant candidates are carefully selected, lung transplantation for SSc-ILD can be performed with minimal risk of exacerbating extrapulmonary manifestations. Evaluating extrapulmonary manifestations based on a protocol may facilitate the selection of appropriate transplant candidates and thus improve the post-transplant outcomes of recipients with SSc-ILD.

The functional benefits obtained from lung transplantation, post-transplant survival, and risks should be considered. Our results suggest short-term improvement and maintenance of pulmonary function after lung transplant for patients with SSc-ILD, as shown in Fig. 1. Additionally, more than 60% of patients ceased long-term oxygen therapy post-transplant.

Bilateral lung transplant is the preferred procedure for patients with CTD-ILD because of the theoretical benefit of improved survival and post-operative right ventricular function and better reserve to compensate for any decline in lung function due to CLAD^34^. Despite evidence supporting the superiority of bilateral lung transplantation, single lung transplants are still utilized in some countries and lung transplant centers^34^. In Japan, bilateral lung transplants are restricted to patients with active chronic infection, severe PH, or refractory pneumothorax due to the shortage of deceased donors. About half of the recipients in our cohort received a single lung transplant for SSc-ILD, with similar post-transplant outcomes between bilateral and single procedures. ILD progression in the native lung was observed over time in one-third of the single lung transplant recipients. Post-transplant changes in the native lung may influence long-term outcomes after single lung transplantation^35,36^. The impact of transplant procedures (bilateral vs single) and ILD progression in the native lung (following a single lung transplant) on post-transplant outcomes should be addressed in a larger cohort.

This study had limitations in terms of generalizability. The number of transplant candidates for SSc-ILD was insufficient to investigate the predictors of fatal outcomes on the waiting list. The high frequency of single lung transplants in Japan may have a significant effect on the waiting time for transplants^37^. A single-institution study design may also have biased the post-transplant outcomes. Although the 2021 ISHLT consensus paper proposed the need for hemodynamic profiles in lung transplant candidates for SSc, only a few patients underwent right heart catheterization pre-transplant^13^.

## Conclusions

Using data from one of the highest-volume lung transplant centers in a non-Western country, we showed that lung transplantation could be an acceptable treatment for selected patients with severe SSc-ILD. Lung transplant indications for SSc and the appropriate time for referral to lung transplant centers should be determined individually in a disease-specific manner.

## Supplementary Information

Supplementary Information.

## Data Availability

The data underlying this article will be shared on reasonable request to the corresponding author.

## Abbreviations

SSc: Systemic sclerosis
ePASP: Estimated pulmonary artery systolic pressure
CLAD: Chronic lung allograft dysfunction
IQR: Interquartile range
DLCO: Diffusing capacity of the lung for carbon monoxide
PFT: Pulmonary function test results
ILD: Interstitial lung disease
HRCT: High-resolution computed tomography
IPF: Idiopathic pulmonary fibrosis
PH: Pulmonary hypertension
ISHLT: International society for heart and lung transplantation
CTD: Connective tissue disease
