# Supplementary material for: Prognosis of patients with systemic sclerosis-related interstitial lung disease on the lung transplant waiting list: a retrospective study
Source: Sci Rep. 2023 Jun 22;13:10150. doi: 10.1038/s41598-023-37141-w (PMC10287698; doi:10.1038/s41598-023-37141-w)
Supplement: Supplementary file 1 — Supplementary Information. [file 41598_2023_37141_MOESM1_ESM.docx]

**Supplementary Figure 1.** Patients with systemic sclerosis-associated interstitial lung disease registered for deceased-donor lung transplants and who underwent lung transplantation.

**
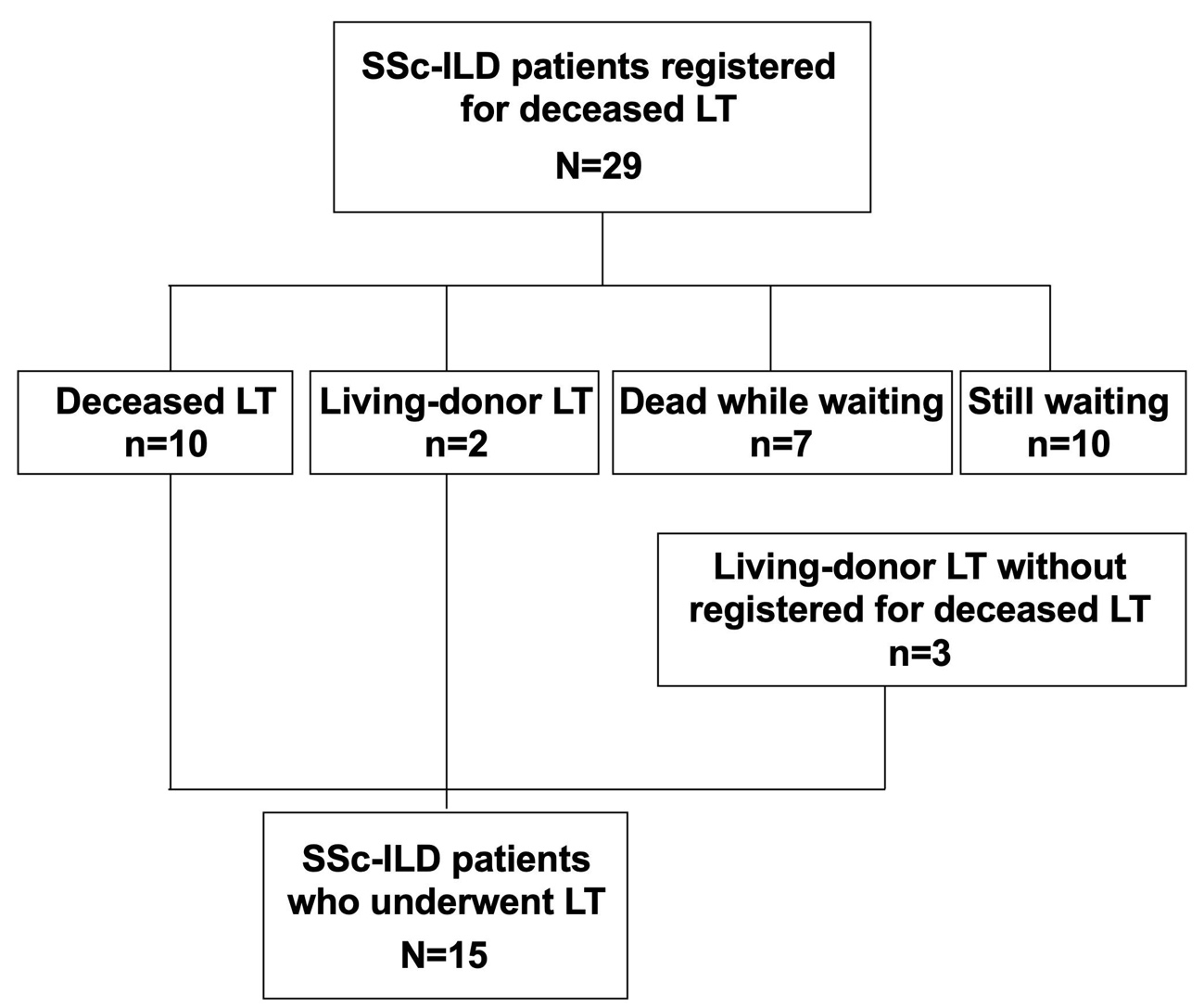
**

**Supplementary Table 1**. Demographic characteristics of SSc-ILD patients who underwent lung transplantation (SSc-ILD with LT) and idiopathic pulmonary fibrosis patients who underwent lung transplantation (IPF with LT).

|  | SSc-ILD with LT (N=15) | IPF with LT (N=20) | *p* value |
| --- | --- | --- | --- |
| Age, mean (S.D.) | 50.4 (11.1) | 55.1 (7.9) | 0.15 |
| Female, n (%) | 10 (67) | 4 (20) | 0.01 |
| BMI (kg/m^2^), median (IQR) | 20.9 [17.7, 24.0] | 20.4 [18.1, 23.4] | 0.87 |
| (Ex-)Smokers, n (%) | 9 (60) | 15 (75) | 0.34 |
| PH^†^, n (%) | 7 (47) | 3 (15) | 0.04 |
| Treatment |  |  |  |
| PSL, n (%) | 13 (87) | 10 (50) | 0.02 |
| PSL dose (mg), median (IQR) | 10 [4, 15] | 14 [8, 18] | 0.46 |
| Calcineurin inhibitors, n (%) | 7 (47) | 4 (25) | 0.27 |
| IVCY (previous use), n (%) | 3 (20) | 1 (5) | 0.17 |
| Spirogram |  |  |  |
| %FVC, median (IQR) | 55.1 [47.8, 60.4] | 41.1 [35.8, 52.1] | 0.13 |
| %DLCO, median (IQR) | 23.7 [20.7, 25.3] | 26.8 [18.7, 34.5] | 0.34 |
| LTOT, n (%) | 12 (92) | 20 (100) | 0.39 |
| Single/Bilateral lung transplantation, n/n | 7/6^‡^ | 11/9 | 0.77 |

^†^ Pulmonary hypertension (PH) was diagnosed if 1) estimated pulmonary artery systolic pressure on transthoracic echocardiogram was 40 mmHg or more, or 2) mean pulmonary arterial pressure on right heart catheterization was 25 mmHg or more.

^‡^ Two patient received single lung transplantation twice.

IVCY, intravenous cyclophosphamide; LTOT, long-term oxygen therapy; PH, pulmonary hypertension; PSL, prednisolone
